# KRDQN: An Interpretable Prediction Framework for Adverse Drug Reactions via Knowledge–Graph Reinforced Deep Q-Learning

**DOI:** 10.3390/ph19030379

**Published:** 2026-02-27

**Authors:** Qiao Ni, Xue Min, Cui Chen, Hongmei Li, Xiaojun He, Linghao Ni, Jiawei Zhou, Bin Peng

**Affiliations:** Department of Health Statistics, College of Public Health, Chongqing Medical University, Chongqing 400016, China

**Keywords:** adverse drug reaction, biomedical knowledge graph, reinforcement learning

## Abstract

**Background:** Adverse drug reactions (ADR) pose substantial risks to patient safety and challenge clinical decision-making. However, traditional predictive approaches frequently fail to deliver interpretable insights into the complex interplay between pharmaceuticals and biological systems. **Methods:** We propose the KRDQN (Knowledge Graph Reinforced Deep Q-Network) predictive framework. First, a knowledge graph (KG) that encompasses five entity types—drug, target, pathway, gene, and adverse drug reaction (ADR)—is constructed, and each node is enriched with intrinsic attribute features. A Deep Q-Network (DQN) is subsequently deployed within a reinforcement learning paradigm to generate interpretable ADR predictions. Model performance is evaluated by five-fold cross-validation, with accuracy and AUC reported. Finally, the Spearman correlation coefficients between drug–drug similarity and path–path similarity are computed, and case studies are conducted to further assess the predictive capability of KRDQN. **Results:** We evaluated KRDQN on a comprehensive data set encompassing both drug–drug interactions and ADR records. Experimental results demonstrate that KRDQN surpasses state-of-the-art baselines, attaining a recall of 0.8171 and an AUC of 0.8327. Furthermore, to demonstrate the practical value of the KRDQN prediction framework, we applied it to predict potential ADRs and their mechanism pathways for the drugs sunitinib and indomethacin. The results indicated that the KRDQN framework could identify biological mechanism pathways consistent with clinical evidence. **Conclusions:** In this study, we developed the reinforcement learning-based KRDQN predictive framework, which outperforms existing baselines in predictive performance and yields interpretable adverse drug reaction (ADR) predictions, thereby serving as a powerful tool for pharmacovigilance and clinical decision-making.

## 1. Introduction

Adverse drug reactions (ADRs) are defined by the World Health Organization (WHO) as any noxious, unintended, or harmful response that occurs at doses normally used in humans and is suspected to be related to drug administration [1]. ADRs endanger patients’ physical and mental health and impose substantial economic losses and societal burdens on healthcare systems. Globally, ADRs account for approximately 800,000 cases of disability or death annually, representing 3.6% of all hospital admissions [2]. In the United States, ADRs constitute the fourth leading cause of death, with more than 100,000 fatalities each year [3]; direct costs attributable to ADRs reach US$ 528.4 billion per annum, equivalent to 16% of total U.S. healthcare expenditure [4]. According to the most recent data from the U.S. Food and Drug Administration (FDA), the number of ADR reports has increased annually. In 2022, the FDA received 2,347,431 ADR reports, of which 53.86% were serious, and 7.46% involved fatal outcomes [5]. In China, over 2.5 million patients are hospitalized annually because of ADRs, resulting in 192,000 deaths [6]. The National ADR Monitoring Network received 2.419 million adverse drug reaction/event reports in 2023, and this figure continues to rise [7]. Following the 2015 reform of the drug review system, the number of newly approved drugs in China has risen sharply. Although these agents undergo rigorous pre-clinical and clinical testing that can reveal toxicity and potential ADRs, statistical studies indicate that detecting ADR incidences between 1/6000 and 1/300 would require surveillance of 10,000–20,000 patients during clinical trials [8]. In practice, limitations in sample size and trial duration render comprehensive pre-marketing ADR detection infeasible [9]. Therefore, predicting potential ADRs, identifying drug–ADR associations, and ultimately reducing ADR incidence are critical for both drug development and clinical pharmacovigilance. Early-stage ADR assessment in drug development primarily relies on toxicological assays; although these yield precise findings, they are labor-intensive and time-consuming [10]. Conventional statistical approaches and clinical trials can likewise predict ADRs, yet they remain costly, protracted, and susceptible to selection and information biases [11]. Consequently, more efficient ADR identification is urgently required. Machine learning offers superior cost-effectiveness and speed relative to traditional methods [12].

Recent advances in machine learning have provided robust technological support for ADR prediction. Nevertheless, most extant studies focus exclusively on prediction and lack computational models specifically designed to explain ADRs; knowledge graph-based approaches can explicitly delineate the pathways through which drugs elicit adverse reactions. Lukashina et al. [13] introduced SimVec, which augments the knowledge graph (KG) structure via structure-aware node initialization and weighted drug similarity edges to predict ADRs in polypharmacy. All state-of-the-art KG-based models for predicting side effects of multi-drug therapy—including RESCAL [14], KBLRN [15], Decagon [16], and TriVec [17]—exploit such knowledge graphs. Despite achieving accurate ADR prediction, these approaches cannot explicate the underlying biological processes. Munoz et al. [18] integrated drugs, diseases, genes, and other biomedical entities into a KG and employed a multi-label learning framework for ADR prediction. Yet, despite leveraging multi-source data, the framework offers no in-depth biological explanation of the predicted ADRs. Wang et al. [19] constructed the Tumor Biomarker Knowledge Graph (TBKG) comprising tumor, biomarker, drug, and ADR nodes, and applied depth-first search (DFS) to enumerate all paths linking drugs to ADRs. However, the explanations provided remain superficial and fail to meet clinicians’ demand for mechanistic insight into ADRs.

In this study, we propose KRDQN, a framework that leverages knowledge graphs and RL for interpretable ADR prediction; it comprises three modules: (i) a multi-layer biomedical KG, (ii) node-feature extraction, and (iii) interpretable ADR prediction. We construct the KG from drug–target, target–pathway, pathway–gene, and gene–ADR relationships, and equip each of the five node types with informative feature vectors—e.g., amino-acid sequences for targets and transcriptomic profiles for genes. Moreover, we combine Deep Q-Networks (DQN) with biologically interpretable “demonstration paths,” employing these paths as reward signals to improve path-searching capabilities within knowledge graphs. While delivering accurate predictions, it also provides biologically plausible interpretive paths.

## 2. Result

### 2.1. Experiment Settings

The KRDQN model was implemented using Python (version 3.12). We utilized the Adam algorithm to optimize all parameters on the dataset with a learning rate (LEARNING_RATE = 0.0008) and an embedding dimension (EMBED_DIM = 32), which helped avoid excessive computational costs and the “curse of dimensionality.” The reward discount factor (GAMMA = 0.946) reflected the model’s focus on long-term rewards. The exploration rate decay (EPSILON_DECAY = 0.987) balanced the exploration and exploitation strategies in the reinforcement learning process. The batch size (BATCH_SIZE = 173) was determined by considering both training efficiency and result stability. All these hyperparameters were finely tuned through multiple preliminary experiments and optimized according to the characteristics of the dataset to achieve the best balance between model convergence speed and predictive performance. Furthermore, to ensure fairness, we re-implemented all baseline methods on the same machine with an embedding dimension of 32.

### 2.2. Baseline Model Comparisons

Baseline models fall into two categories: graph embedding-based methods (e.g., GCN and DeepWalk) and path-based methods (e.g., LSTM and KPRN). In adverse drug reaction prediction tasks, these baseline models were compared in terms of performance. The results are presented in Table 1. Results show that the KRDQN model outperforms all baselines in AUC and F1 metrics. While its advantages in other metrics like accuracy, precision, and recall are relatively small, the KRDQN model demonstrates significant superiority over other baselines due to its explainability.

When comparing explainability performance across graph-based methods, the results are presented in Table 2. KRDQN exhibits negative fidelity values at target, pathway, and gene levels (target = −1.28009, pathway = −0.442875, gene = −0.645055), markedly contrasting with traditional non-reinforcement learning models (DeepWalk, Node2Vec, and GNN) that yield positive values. From an interpretability perspective, this “negative fidelity” reflects the model’s sensitivity to nodal structural heterogeneity and functional responsiveness. Particularly at gene and pathway levels, the reinforcement learning strategy actively explores unconventional pathways during reward signal optimization, thereby revealing potential reverse regulatory mechanisms or compensatory pathways. This characteristic demonstrates that KRDQN does not merely replicate known mechanisms but learns dynamic patterns of inter-nodal differentiation within the search space, conferring superior mechanistic resolution capacity.

### 2.3. Five-Fold Cross-Validation

**Figure 1:**
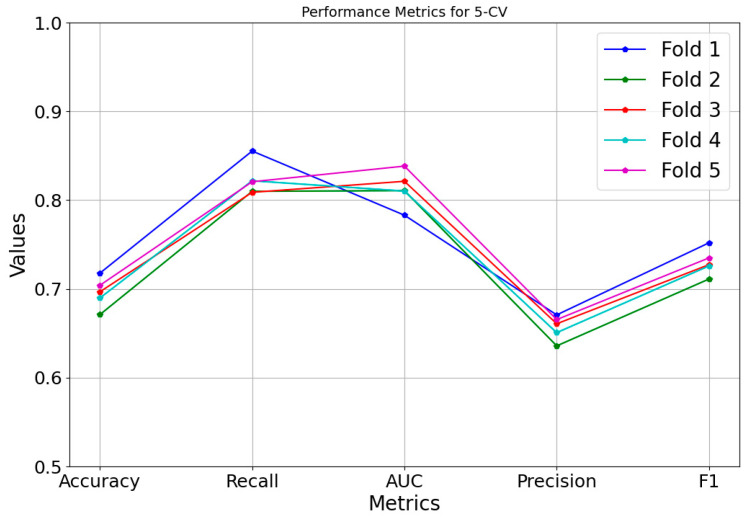
Performance of each metric across the five folds of cross-validation.

To demonstrate model stability, we visualized the results of each fold in the 5-fold cross-validation for accuracy, precision, and other metrics; as illustrated in Figure 1, the nearly overlapping trajectories across folds indicate consistent performance across diverse data subsets.

### 2.4. Ablation Study

We conducted systematic ablation studies on the key components of the proposed model, with results presented in Figure 3. Overall, the performance impact (reward, accuracy, F1) of varying parameter settings remains relatively modest, indicating strong robustness of the model to certain hyperparameter and architectural design choices.

**Figure 2:**
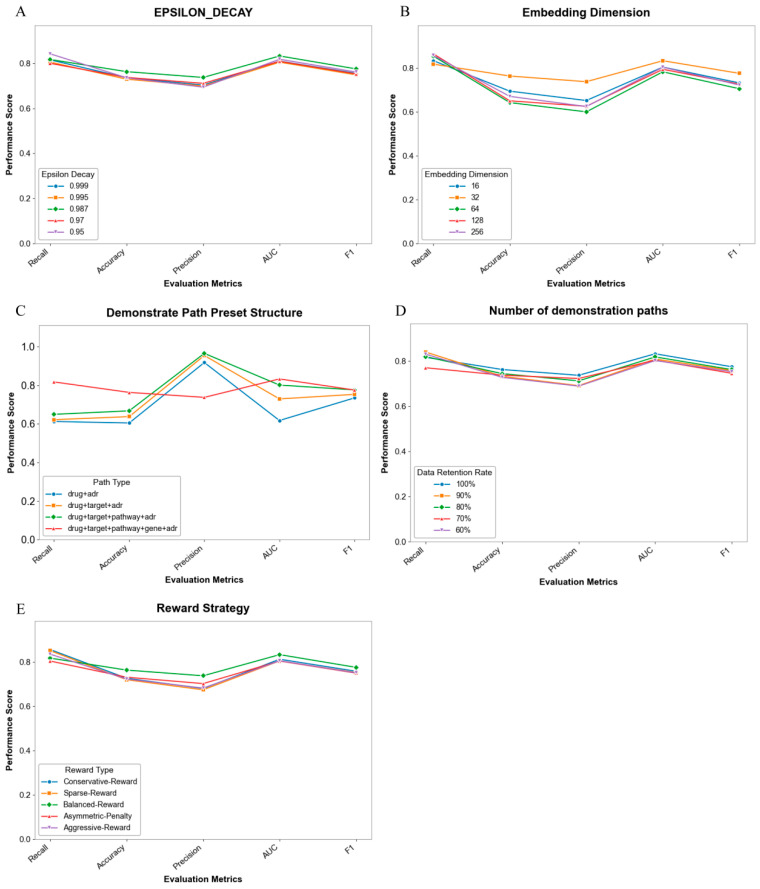
Ablation study results of key model components and parameters. (**A**) Performance changes under different EPSILON_DECAY values. (**B**) Model performance with different embedding dimensions. (**C**) Impact of four different demonstration path configurations on model performance. (**D**) Effect of the number of demonstration paths on model performance. (**E**) Performance comparison of the model under different reward strategies. In all subplots, the x-axis shows performance metrics (reward, accuracy, F1), the y-axis shows the corresponding scores, and there is clear visual differentiation for parameter comparisons.

Specifically, for the EPSILON_DECAY parameter (Figure 2A), lower decay values (e.g., 0.95) enhance initial exploration adequacy but compromise long-term stability; conversely, higher decay values (0.998) improve convergence stability at the cost of reduced exploration efficiency. Regarding embedding dimensions (Figure 2B), a dimensionality of 32 achieves an optimal trade-off: insufficient representation capacity emerges at lower values (16), while redundant noise is introduced at higher values (256). Furthermore, with respect to demonstration path preset structures (Figure 2C), reasonable incorporation of structural priors significantly enhances path planning rationality, wherein the hierarchical structure variant improves the F1 score by approximately 3.2%. Concerning demonstration path quantity (Figure 2D), retaining 80% of paths maintains performance while improving training efficiency; retaining excessive paths (100%) tends to induce overfitting, whereas retaining too few (50%) leads to insufficient exploration. Finally, in comparative reward strategy experiments (Figure 2E), the Balanced-Reward strategy, albeit suboptimal on recall metrics, significantly outperforms alternatives on Precision and F1 score. These findings provide empirical evidence for configuring model key components, and subsequent experiments will select optimal parameter combinations based on the aforementioned discoveries.

**Figure 3:**
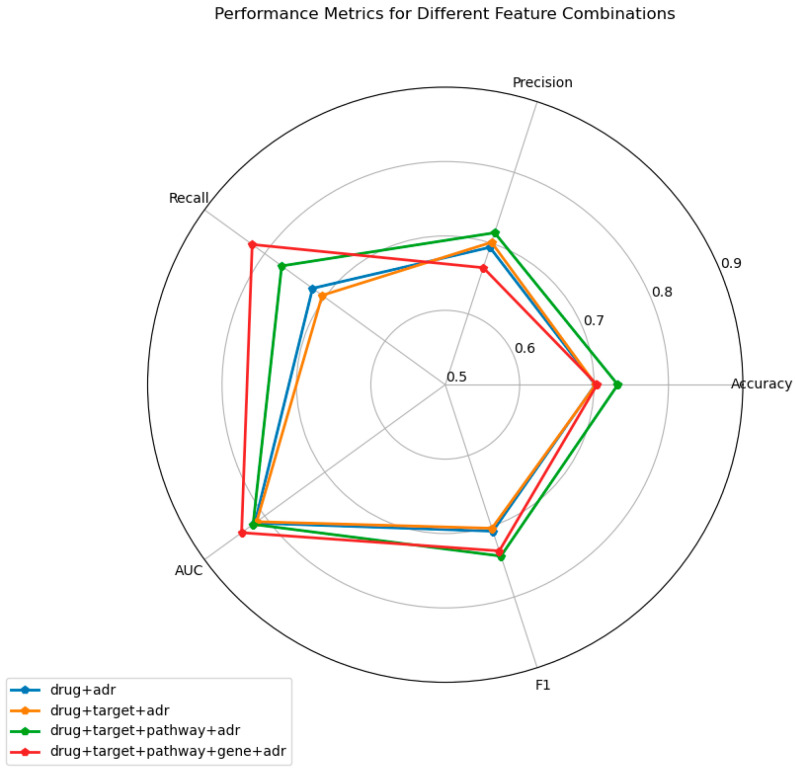
Variation in model performance across different feature-combination settings.

To demonstrate the effectiveness of incorporating multi-modal features for adverse drug reaction (ADR) prediction, we systematically evaluated model performance under different feature combinations.The results are depicted in Figure 3. Four configurations were examined: (i) drug + ADR, (ii) drug + target + ADR, (iii) drug + target + pathway + ADR, and (iv) drug + target + pathway + gene + ADR. All models were trained and assessed on an identical test set.

As the feature set becomes more comprehensive, a consistent upward trend is observed across all evaluation metrics. When only the drug + ADR combination is employed, the model yields comparatively low accuracy, precision, recall, AUC, and F1-score. Sequential inclusion of target, pathway, and gene information yields substantial improvements, most notably in AUC (from 0.8163 to 0.8327) and sensitivity (from 0.7204 to 0.7629). The configuration incorporating all available features significantly outperforms the remaining combinations, underscoring that richer pharmacological representations enhance predictive capability and highlighting the critical importance of multi-modal feature fusion.

### 2.5. Explain Performance Evaluation

**Figure 4:**
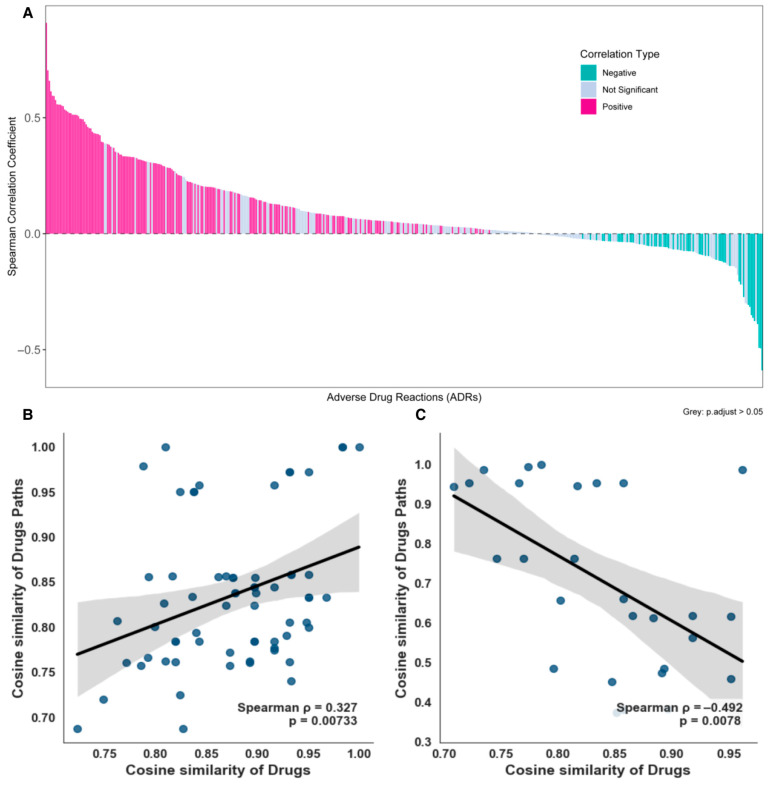
(**A**) Distribution of Spearman correlation coefficients between drug–drug similarity and biomedical pathway–pathway similarity. (**B**,**C**) The relationship between the similarity of drugs and the similarity of paths.

After imposing a sample size threshold of at least 8 drugs per ADR, we analyzed 468 ADR-specific datasets to calculate drug–drug and pathway–pathway cosine similarities. As shown in Figure 4A, 235 datasets exhibited statistically significant correlations (assessed via Spearman’s ρ with Benjamini-Hochberg-adjusted *p* < 0.05). Among the significant datasets, 175 showed positive correlations, while 60 demonstrated negative correlations. Overall, 175 datasets (74.5% of significant cases) had Spearman’s ρ > 0, indicating that the KRDQN framework effectively captures the complex biological relationships between drugs and pathways in most statistically significant instances. Figure 4B,C illustrate the relationship between drug similarity and pathway similarity for the adverse drug reactions (ADRs) “Wound” and “Hepatitis B”.

### 2.6. Analysis of Class Imbalance Impact on Rare ADR Prediction

**Figure 5:**
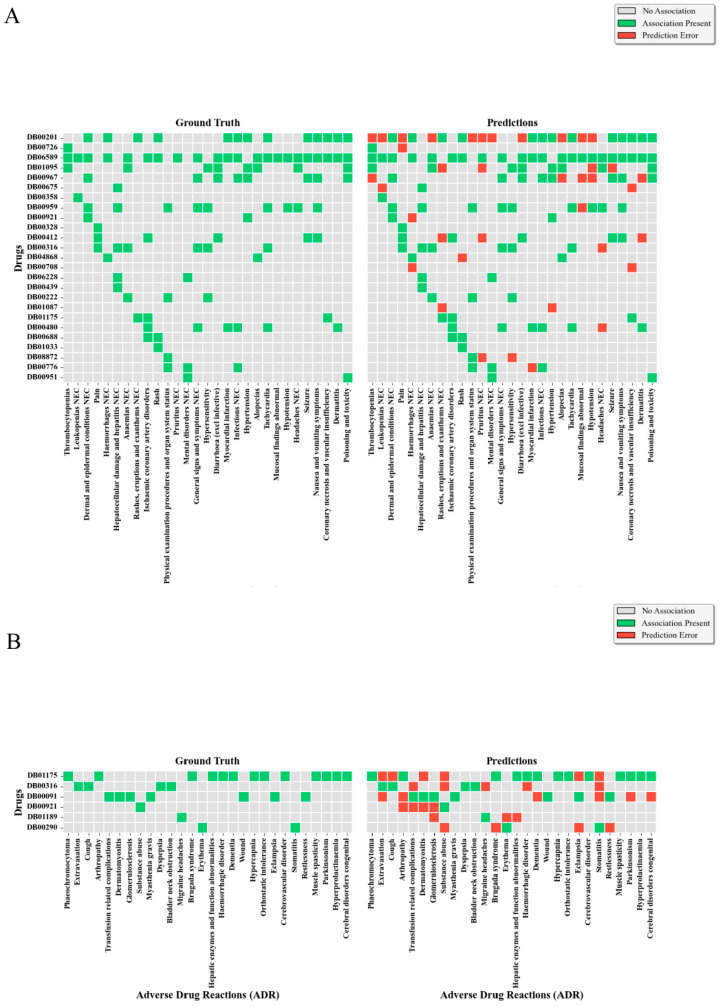
(**A**) KRDQN model predictions versus ground truth for the most common 5% of adverse drug reactions (ADRs). Drug IDs are labeled on the vertical axis and ADR names on the horizontal axis. The left heatmap shows actual outcomes, the right heatmap shows predicted outcomes, with red regions indicating prediction errors. (**B**). KRDQN model predictions versus ground truth for the rarest 5% of adverse drug reactions (ADRs).

To address the severe class imbalance issue inherent in ADR datasets, this study systematically evaluated the predictive robustness of the KRDQN model under extreme frequency distributions. Specifically, the test set was partitioned into two subsets: high-frequency ADRs (top 5%, designated as the common group) and low-frequency ADRs (bottom 5%, designated as the rare group), with separate confusion matrices constructed to quantify performance disparities between the two cohorts. As illustrated in Figure 5, for the common ADR group (encompassing 725 drug–ADR pairs across 29 ADR types and 25 drugs), the model correctly predicted 103 true positive associations (green zone) while misclassifying 38 associations (red zone), achieving a precision of 73.05%. In contrast, for the rare ADR group (encompassing 168 drug–ADR pairs across 28 ADR types and 6 drugs), KRDQN correctly identified 28 true positive associations and misclassified 28 associations, yielding a precision of only 50%. Notably, despite the lower absolute error count in the rare group, its precision (50%) remains substantially lower than that of the common group (73.05%), which corroborates the inherent challenges of learning from sparse samples.

### 2.7. Case Study

To explore whether the interpretability pathways identified by the KRDQN prediction framework align with clinical evidence, we visually presented the biological pathways of sunitinib-induced alopecia and indomethacin-induced hepatitis.

**Figure 6:**
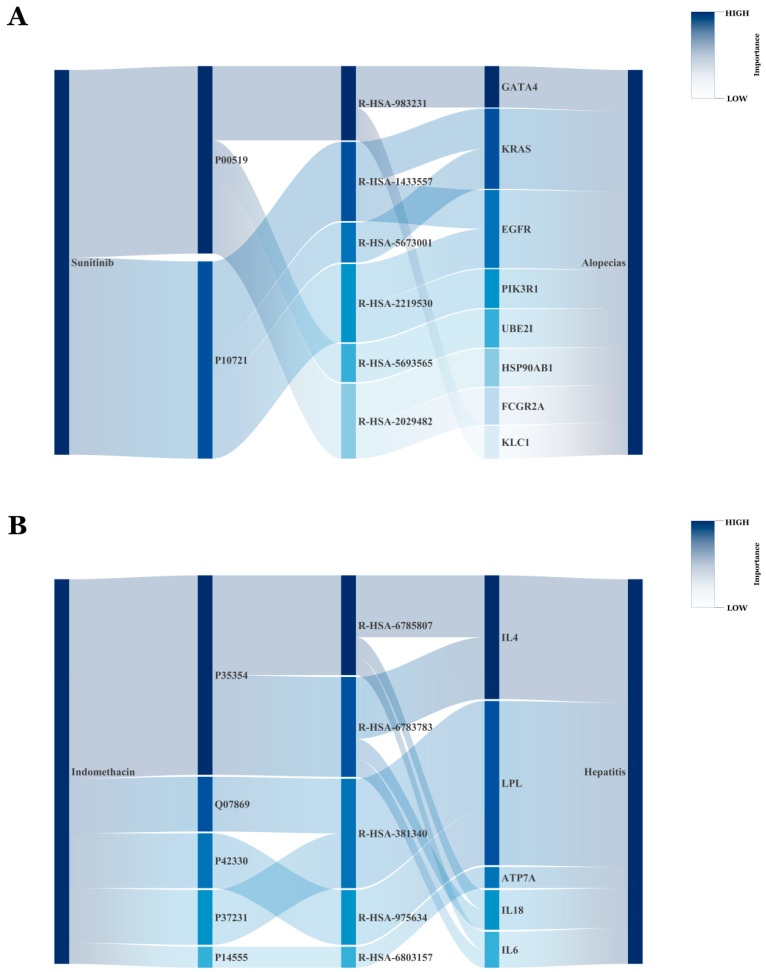
Presents alluvial diagrams that delineate the principal pathways connecting sunitinib to alopecias (**A**) and indomethacin to hepatitis (**B**). The width of each ribbon is proportional to the magnitude of the corresponding q-value, whereas darker shading of nodes signifies elevated importance.

Figure 6A depicts the ten most probable pathways through which sunitinib (DB01268) induces alopecias. Sunitinib-induced alopecia is closely associated with inhibition of specific target kinases and signaling pathways, consistent with its activity as a multi-target tyrosine kinase inhibitor [20]. Clinical evidence demonstrates that sunitinib-mediated hair loss primarily involves inhibition of key targets including vascular endothelial growth factor receptors (VEGFR; e.g., P10721) and platelet-derived growth factor receptors (PDGFR) [21]. These receptors regulate homeostasis of the hair follicle microenvironment, wherein VEGF pathway inhibition (Reactome R-HSA-1433557) disrupts follicular angiogenesis and cell survival signaling [22]. Additionally, sunitinib-mediated c-KIT inhibition impairs melanocyte function within the follicular pigmentary unit, resulting in clinically observed hair depigmentation [23]. Although downstream effectors including KRAS, EGFR, and PIK3R1 are within sunitinib’s inhibitory spectrum, direct evidence implicating these pathways in alopecia pathogenesis remains to be established. Long-term follow-up data from the Phase III INTRIGUE trial corroborate the association between sunitinib treatment and alopecia as a specific adverse event, despite no statistically significant difference in overall survival compared with control therapy. Case series have documented early-onset alopecia during sunitinib therapy (median onset: 4–6 weeks) [24]. While this alopecia is typically reversible, the underlying molecular mechanisms—including potential roles for transcription factors such as GATA4—require further investigation.

Figure 6B illustrates the ten most probable pathways through which indomethacin (DB00328) causes hepatitis. The core mechanism underlying indomethacin-induced drug-induced liver injury originates from its aberrant direct activation of peroxisome proliferator-activated receptor α (PPARα) and γ (PPARγ) [25]. Studies have demonstrated that indomethacin functions as a ligand to directly bind and activate PPARα/γ, and this aberrant activation significantly upregulates the expression of downstream lipid metabolism-related genes; lipoprotein lipase (LPL), serving as a key target gene, promotes the influx of massive free fatty acids into hepatocytes and their subsequent conversion into triglyceride deposition, ultimately inducing hepatic steatosis [26,27]. Severe lipid accumulation within hepatocytes can further elicit metabolic stress, including endoplasmic reticulum stress and oxidative stress; experimental evidence has confirmed that following indomethacin treatment of hepatocytes, the expression levels of endoplasmic reticulum stress markers (such as phosphorylated eukaryotic translation initiation factor 2α (p-eIF2α), C/EBP homologous protein (CHOP), and spliced X-box binding protein 1 (XBP1s)) increase in a time-dependent manner, concurrently activating apoptosis-executing proteins including cleaved caspase-3 [28], ultimately triggering hepatocyte apoptosis and inflammatory responses, thereby leading to hepatitis [29].

**Figure 7:**
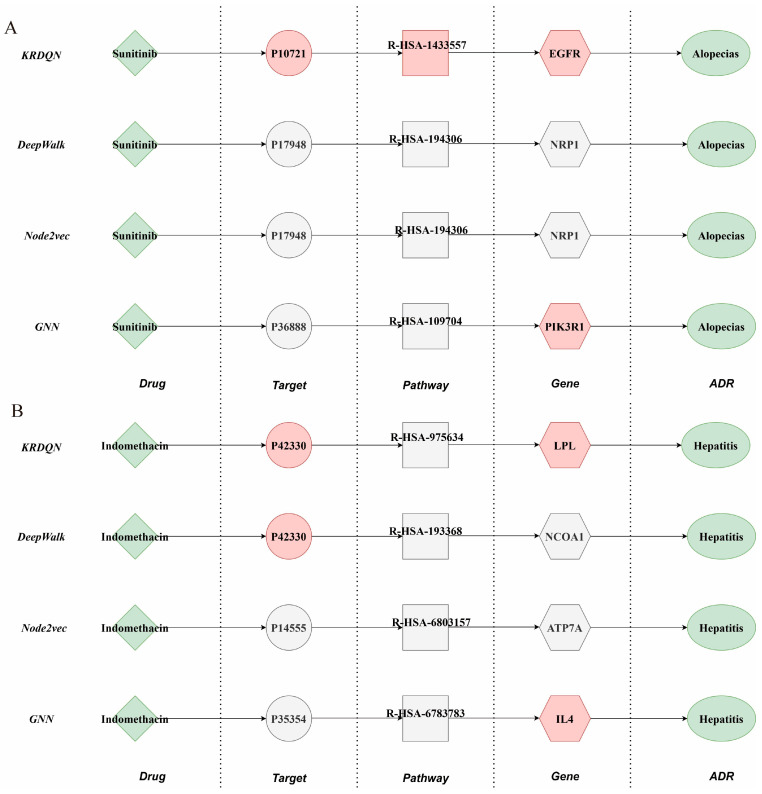
Comparison of the interpretability paths generated by the KRDQN model and three baseline models (DeepWalk, Node2Vec, and GNN) for two drug–adverse reaction pairs. Panel (**A**) illustrates the interpretability paths linking Sunitinib and Alopecias, while panel (**B**) shows those linking Indomethacin and Hepatitis.

In the alopecias task, pathways identified by the KRDQN model demonstrated high concordance with authentic biological mechanisms, as shown in Figure 7A. Drug-induced inhibition of the EGFR signaling pathway leads to impaired hair follicle regeneration, which aligns with clinical manifestations of alopecia. By contrast, the DeepWalk and Node2Vec models only captured the ancillary NRP1 pathway, while the PIK3R1 signal from the GNN model exhibited weaker relevance, underscoring the superior credibility of KRDQN in elucidating drug-alopecia mechanisms.

In the hepatitis task, the LPL pathway discovered by the KRDQN model closely matched mechanisms of drug-induced hepatitis and hepatic lipid metabolism dysregulation, as illustrated in Figure 7B; both the ATP7A pathway from the Node2Vec model and the IL4 pathway from the GNN model showed weak association. Although DeepWalk generated the identical LPL pathway, it lacked sufficient mechanistic explanatory depth.

Collectively, the key pathway nodes identified by the KRDQN model in both drug-phenotype tasks exhibited strong alignment with clinical and molecular evidence. While incurring increased model complexity, KRDQN substantially enhanced the biological interpretability and mechanistic precision of the generated pathways.

## 3. Discussion

The generalization capacity is crucial for adverse drug reaction (ADR) prediction. The KRDQN model integrates a biomedical knowledge graph with reinforcement learning, thereby attaining high accuracy while simultaneously enabling transparent mechanistic reasoning. Compared with conventional baselines, KRDQN yields superior predictive performance; five-fold cross-validation produces stable ROC curves that corroborate its strong generalisability and align with the widely adopted stability-assessment protocol [30], collectively confirming the model’s reliability. Systematic experiments demonstrate that parameter settings including exploration parameters, demonstration path structure, number of demonstration paths, and reward strategy had no significant effect on the model, and embedding dimensionality significantly influences performance, with an optimal peak observed at d = 32. Although higher dimensions can capture finer-grained patterns and modestly improve accuracy, they simultaneously incur the curse of dimensionality [31]; consequently, dimensionality must be selected judiciously. Incorporating the complete feature set—drug, target, pathway, gene, and ADR—maximizes predictive performance, underscoring the necessity of comprehensive feature integration [32].

PCA analysis reveals gene nodes retain 85.67% variance with 32 components, while pathway nodes retain only 40.13%. This disparity reflects their feature extraction methods: Gene features (3-mer frequency) are structurally regular and stable, enabling effective low-dimensional representation; pathway features (TF-IDF matrices) capture complex biological semantics across sparse, high-dimensional space, dispersing variance across more components. Future work should increase component count or adopt a non-linear reduction to better preserve pathway information.

Although the KRDQN framework demonstrates robust overall performance, its precision for rare adverse drug reactions (ADRs) remains notably lower than that for common ADRs. As detailed in Section 2.6, the precision declined from 73.05% in the common ADR group to 50% in the rare ADR group. This disparity highlights the inherent challenge of learning from sparsely represented classes in pharmacovigilance data. Rare ADRs are often the most clinically severe but occur too infrequently for the model to capture distinguishing patterns, leading to limited discriminative power and higher uncertainty in prediction. This limitation primarily stems from class imbalance in existing ADR repositories—datasets contain abundant samples of well-known, frequently reported reactions, while low-frequency but critical events remain underrepresented. As a result, model optimization tends to favor majority patterns, inadvertently biasing predictions toward common ADRs, and conventional loss functions treat all misclassifications equally, lacking sensitivity to rare but high-risk cases.

Moreover, the significant positive correlation between drug–drug similarity and biological-pathway similarity demonstrates that KRDQN predictions are mechanistically interpretable. Among the 235 ADR sets that reached statistical significance, 175 exhibited a positive correlation between pathway similarity and culprit-drug similarity (Benjamini–Hochberg-adjusted *p* < 0.05). This finding is consistent with extensive literature showing that chemical similarity serves as a robust proxy for shared mechanisms: structurally related drugs are significantly more likely to share targets, pathways, and adverse outcomes, indicating that KRDQN faithfully recapitulates complex biomedical relationships [33,34,35]. Conversely, 60 ADR sets displayed a significant negative correlation, underscoring that high structural similarity does not guarantee mechanistic similarity—structure–activity relationship (SAR) cliffs, polypharmacology paradoxes, and scaffold-hopping studies have repeatedly demonstrated that closely related analogues can elicit divergent or even opposite mechanisms [36,37,38]. Case-by-case inspection further revealed that the core pathways identified by KRDQN for pravastatin-induced hypertension and indomethacin-induced hepatitis are in strong agreement with published clinical evidence.

Nevertheless, the model exhibits several limitations. First, the static ADR database may fail to capture newly emerging reactions, resulting in misclassification of new or sparsely annotated drugs; consequently, continuous database updates are required to maintain integrity and timeliness. Second, class imbalance may bias the model toward the majority class. Third, the current representation of protein targets relies primarily on amino-acid composition and averaged physicochemical attributes, which may overlook crucial structural and functional determinants governing drug–target and off-target interactions—such as binding-site topology, conformational dynamics, and catalytic-pocket geometry. Future work should explore resampling strategies and cost-sensitive or weighted loss functions to enhance sensitivity to minority classes.

## 4. Materials and Methods

### 4.1. Data Processing

#### 4.1.1. Data Sources

To assemble the KG, drug–target interactions were retrieved from DrugBank [39], target-involved pathways from Reactome [40], and downstream genes within these pathways from Pathway Commons [41]; only transcriptional responses (i.e., up- or down-regulation of downstream genes) were considered. ADRs and their associated genes were subsequently obtained from ADReCS-Target [42].

Node attribute features were compiled from three public repositories: DrugBank supplied SMILES notations for 213 approved small-molecule drugs; UniProtKB/Swiss-Prot provided amino-acid sequences for 549 corresponding target proteins; and NCBI hosted FASTA-formatted transcript sequences for 601 genes.

To train the KRDQN framework for interpretable adverse drug reaction prediction, 18,559 high-confidence drug–ADR pairs were extracted from the ADReCS database after rigorous filtering and randomly split into training (80%) and testing (20%) sets for subsequent model development and evaluation.

#### 4.1.2. Data Partitioning

To establish a balanced dataset and ensure unbiased model evaluation, for each positive drug–ADR pair, we randomly selected one adverse reaction that was not recorded for the same drug within the ADReCS database to construct a corresponding negative sample.

We employed a drug-level data-splitting strategy to avoid potential data leakage across the training and testing sets. After data cleaning, a total of 208 drugs maintained complete connectivity with adverse drug reactions (ADRs) in the knowledge graph. We divided these drugs into training and testing sets according to an 8:2 ratio, resulting in 166 drugs in the training set and 42 drugs in the testing set. This design ensured that no drug appeared simultaneously in both sets, thereby effectively preventing information leakage and enabling a fair evaluation of the model’s generalization capability to unseen drugs.

### 4.2. Overview of the Predictive Methodology

**Figure 8:**
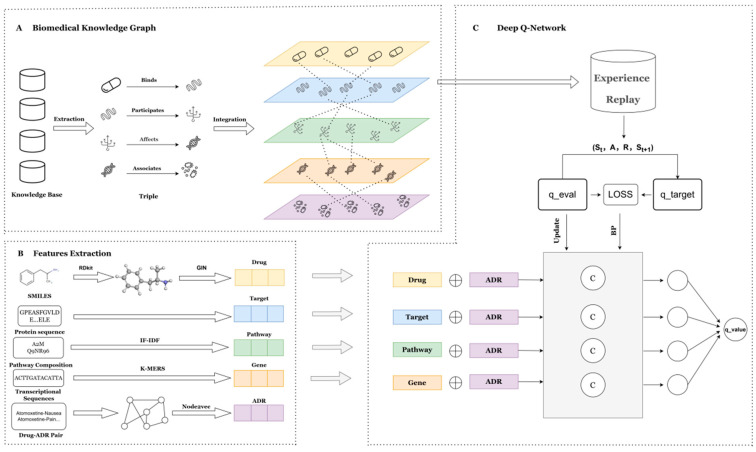
Schematic overview of the proposed technical pipeline. (**A**) Biomedical knowledge graph module. (**B**) Feature extraction module: assigns biologically relevant attribute features to every node in the knowledge graph. (**C**) Explainable ADR prediction module: employs a DQN model to predict ADRs and generate corresponding interpretable pathways.

The KRDQN framework is a deep-learning approach for mechanistic pharmacovigilance that is primarily grounded in the Deep Q-Network (DQN) paradigm of reinforcement learning. The predictive framework comprises three sequential modules, as illustrated in Figure 8.

(1) a biomedical knowledge graph that extracts candidate pathways for subsequent model training.
(2) a feature extraction module that acquires biologically meaningful embeddings for every biomedical entity.
(3) a DQN model that generates explanatory pathways and corresponding q-values for each drug–ADR pair in the network, thereby quantifying the statistical validity of each pathway.

### 4.3. Biomedical Knowledge Graph

#### 4.3.1. Tailored Biomedical Knowledge Graph

To accurately predict adverse drug reactions (ADRs) and provide mechanistic insights, an optimal ADR knowledge graph (KG) must integrate comprehensive biomedical knowledge from diverse databases and publications [43] and explicitly reconcile heterogeneous identifiers denoting identical biological entities. In this graph, every biological concept is represented as a node, and every concept–predicate–concept relationship is encoded as an edge. To enhance predictive precision, we refined the KG according to three principled criteria: (i) removal of low-confidence edges based on predefined quality metrics; (ii) elimination of redundant edges; and (iii) deletion of orphaned nodes lacking a complete path to any ADR node (e.g., drugs disconnected from downstream ADRs). After these pruning steps, our tailored KG comprised 3033 nodes spanning five distinct entity types and 15,272 edges, which were subsequently employed for downstream model training.

#### 4.3.2. Extraction of Demonstrative Pathway

To incentivize the reinforcement learning agent to terminate path exploration at the target adverse drug reaction (ADR) via biologically plausible trajectories, we introduce demonstration paths that mechanistically elucidate how a drug may elicit the reaction. A set of biologically feasible paths—exemplified by drug → target → pathway → gene → ADR—was predefined to ensure both comprehensiveness and structural consistency, obliging the agent to initiate traversal at a drug node, transit via three intermediary node types (target, pathway, gene), and conclude at an ADR node. Consequently, 273,602 demonstration paths were extracted from the curated biomedical knowledge graph and reserved for downstream model training.

### 4.4. Feature Extraction Module

#### 4.4.1. Drug Feature Extraction

These SMILES strings were converted into RDKit molecule objects using the RDKit toolkit. Extended-connectivity fingerprints (ECFPs) were subsequently generated via the AllChem.GetMorganFingerprintAsBitVect function in RDKit, with a radius of 2 and a bit-vector length of 32. ECFPs encode atom-centered structural environments, thereby capturing salient molecular features; the radius parameter governs the neighborhood size, while the bit-vector dimensionality determines the feature–space resolution.

#### 4.4.2. Target Feature Extraction

Drug targets are essentially constituted by amino-acid sequences, which serve as the basis of their molecular identity and structural framework. For each sequence, we first computed the absolute counts of the 20 standard amino acids (A, C, D, E, F, G, H, I, K, L, M, N, P, Q, R, S, T, V, W, Y) and then derived their relative frequencies. These 20 frequency values constitute the first 20 dimensions of the feature vector and capture the amino-acid compositional profile of each target. We further summarized 12 physicochemical properties—including hydrophobicity, volume, and polarity—across the entire sequence. The mean value of each property across the sequence was calculated, yielding the remaining 12 dimensions. The resulting 20 amino-acid frequencies and 12 physicochemical means were concatenated to produce a feature vector for each target.

#### 4.4.3. Pathway Feature Extraction

A biological pathway constitutes an ordered cascade of genes, proteins, or metabolites with distinct functions that collectively accomplish a specific biochemical process. We retrieved pathways containing the targets from Reactome and extracted their downstream genes from Pathway Commons. The associated targets and genes were concatenated into a unified textual representation for each pathway. TF-IDF was applied to quantify the importance of each target or gene within a given pathway. A TF-IDF matrix was constructed with 695 rows (pathways) and columns corresponding to unique targets or genes; entries denote TF-IDF scores that down-weight ubiquitous entities while highlighting pathway-specific components. Because the original TF-IDF matrix is high-dimensional—its feature count equals the number of unique targets and genes—direct usage would raise computational costs and risk overfitting. Principal component analysis (PCA) was therefore employed to reduce the dimensionality to yield compact pathway embeddings.

#### 4.4.4. Gene Feature Extraction

Each transcript was decomposed into overlapping 3-mers using a sliding-window k-mer algorithm (e.g., “ACGT” → [“ACG”, “CGT”]). Absolute frequencies of each 3-mer were computed and normalized to relative frequencies, resulting in a high-dimensional feature vector where each dimension represents the frequency of a unique 3-mer. PCA was subsequently applied to reduce the dimensionality of the resulting feature space.

#### 4.4.5. ADR Feature Extraction

To derive biologically meaningful ADR feature vectors, we first identify all ADR nodes within the previously constructed multi-layer biomedical heterogeneous network. Subsequently, the Node2Vec algorithm is employed, leveraging a random walk strategy that favors depth-first search. Based on the generated node sequences, low-dimensional node representations are learned using an approach akin to Word2Vec. Ultimately, by constructing a heterogeneous graph and applying the Node2Vec algorithm, ADR feature vectors that encapsulate complex biomedical relationships are successfully generated.

### 4.5. Deep Q-Network Model

#### 4.5.1. Experience Replay Module

In reinforcement learning, the agent–environment interaction is inherently sequential, so consecutively sampled states and rewards exhibit strong temporal correlations. Such temporal dependencies can bias gradient estimates and thereby compromise convergence. Experience replay mitigates this issue by storing historical transitions in a replay buffer and subsequently sampling them uniformly at random, effectively decorrelating the data. Training on sequentially acquired data further destabilizes learning because contiguous transitions often share similar features or reward distributions. By sampling mini-batches uniformly, experience replay ensures diverse training data at each update step, enhancing both stability and convergence speed.

We therefore store each demonstrated pathway as an experience tuple (state, action, reward, next_state, target_ADR) in the replay buffer. During training, a mini-batch of tuples is randomly drawn from this buffer to update the parameters of the Q-network.

#### 4.5.2. Deep Q-Network

In the context of adverse drug reaction (ADR) prediction, the neural network receives a state representation and outputs action-value estimates for all admissible actions. Traditional Q-learning associates states with actions via a tabular Q-table; in contrast, DQN parameterizes this mapping with a neural network to approximate the cumulative return.

(1) Input layer:

All entity types—drugs, targets, pathways, genes, and adverse drug reactions—are embedded into a shared 32-dimensional latent space via learnable embedding matrices. Formally, the embedding of node i is denoted by e_i_ ∈ R^32^. Because the agent traverses the knowledge graph in discrete hops, we additionally introduce step embeddings e_s_ ∈ R^32^ to encode the current hop index.

(2) Hidden layers:

The node, step, and target ADR embeddings are concatenated to form the input vector *h* = [e_i_; e_s_; e_adr_] ∈ R^96^. This vector is fed into a stack of fully connected layers with ReLU activations. Let W(l) and *b*(l) denote the weight matrix and bias vector of layer1, the forward pass is computed as:

(1) $$a\left(l\right)=ReLU\left(W\left(l\right)h(l-1)+b\left(l\right)\right)$$

Specifically, *h*(*l* − 1) denotes the output vector of layer(*l *− 1), which serves as the input to layer l. Consequently, for the first hidden layer, the input vector corresponds to the concatenated representation *h* defined previously. Dropout regularization is applied to the hidden layers to mitigate overfitting. During training, dropout randomly deactivates a subset of neurons, thereby preventing the model from relying excessively on specific units and enhancing its generalization capability.

(3) Output layer:

It produces a scalar *Q*-value *Q*(s, a) for each admissible action in a given state s. Let o ∈ R^H^ denote the final hidden representation, where H is the hidden dimension. The *Q*-value is then computed as:

(2) $$Q=W\left(out\right)o+b\left(out\right)$$

This value represents the expected cumulative reward for transitioning from the current node to the target ADR under the given action; a higher *Q*-value thus indicates a more promising action toward the desired ADR.

During path search, the agent receives intermediate rewards as well as a terminal reward upon reaching the target. Intermediate rewards guide incremental progress, whereas the terminal reward provides positive feedback upon successful arrival. However, relying solely on positive rewards fails to penalize undesirable states. Consequently, negative rewards are introduced to explicitly discourage incorrect states, steering the agent toward valid trajectories. To clarify the definition of a valid path during reinforcement learning, we further constrained the agent’s exploration using biologically validated demonstration paths. These demonstration paths serve as structural priors extracted from the biomedical knowledge graph, comprising drug–target–pathway–gene–ADR relationships supported by curated domain knowledge. Consequently, the agent’s action space is restricted to transitions within these biologically plausible structures rather than arbitrary graph connections.

During navigation, when the agent performs consecutive node transitions that remain within the hierarchical structure of the demonstration paths, it is considered to be “maintaining an effective pathway” and is rewarded with +1 as an intermediate incentive. Deviations from these structural constraints result in the loss of the intermediate reward or incur a penalty (−5). This biologically grounded reward design ensures that the exploration process aligns with realistic pharmacological mechanisms, improving both the interpretability and reproducibility of the RL framework:

(3) $$R\left\{\begin{array}{c} 5,if\;vt=a\;\\ 1,if\;vt=V\\ -5,if\;vt\neq a \end{array}\right.$$

### 4.6. Training Procedure

#### 4.6.1. Problem Formulation and Experience Storage Structure

The drug–ADRs association prediction task was formulated as a Markov decision process (MDP) defined by the tupleState: S_t_ = (V_t_,k,a_target_), where V_t_ denotes the current node, k ∈ {0, 1, 2, 3} counts the completed hops, and atarget is the atarget node; Action space: A consists of all one-hop neighbors reachable from V_t_; Reward function: r = +5 if the agent reaches the target ADR within k = 3 hops, +1 for any intermediate step that remains on a valid path, and −5 otherwise; Termination: episodes terminate upon reaching k = 4 or when no valid actions remain. To mitigate issues arising from temporal correlations and non-stationary distributions, we employed experience replay. Each transition tuple Et = (S_t_, a_t_, R_t_, S_t+1_, done) was stored in a uniform replay buffer with capacity 10^6^.

#### 4.6.2. Target Q-Value Computation

During training, mini-batches of size B = 173 were sampled uniformly and at random from the replay buffer to guarantee sample independence. The policy network Q was updated using one-step temporal-difference (TD) error; for each transition in the mini-batch, the target Q-value was computed according to Equation (4):

(4) $$Q_{target}(s^{\prime },a^{\prime })=R+\gamma \cdot Q_{target}\left(s,a\right)\cdot (1-done)$$

#### 4.6.3. Loss Function and Gradient Update

The training objective is to minimize the mean-squared Bellman error (MSBE):

(5) $$L(\theta )=\frac{1}{B}\sum_{i=1}^{B}{\left(Q_{eval}-Q_{target}\right)}^{2}$$

Gradient descent was performed with the Adam optimizer (initial learning rate η = 8 × 10^−4^). To mitigate overfitting, dropout with a probability of *p* = 0.3 was applied between all fully connected layers.

#### 4.6.4. Exploration-Annealing Strategy

An ε-greedy policy was employed, with ε initialized to 1.0 and decayed exponentially by a factor of 0.987 per episode until a minimum of 0.066 was reached. Consequently, the agent progressively shifted from random exploration to Q-value-guided exploitation, enhancing policy stability.

#### 4.6.5. Learning Rate Update

After every episode, the learning rate scheduler was invoked to update the learning rate. At the end of each episode, the current model parameters were saved to disk.

### 4.7. Evaluation Metrics

In this study, we employ five widely adopted metrics—accuracy, precision, F1-score, and the area under the receiver operating characteristic curve (ROC-AUC)—to evaluate model performance. The formal definitions of these metrics are provided below.

(6) $$Accuracy=\frac{TP+TN}{TP+TN+FP+FN}$$

(7) $$Precision=\frac{TP}{TP+FP}$$

(8) $$Recall=\frac{TP}{TP+FN}$$

(9) $$F1=\frac{2\times Precision\times Recall}{Precision+Recall}$$

### 4.8. Interpretability Assessment

To assess the interpretability of KRDQN predictions, we computed the cosine similarity between drugs and their associated pathways under the same adverse drug reaction (ADR). To ensure robustness, only ADR-specific drug–pathway sets containing more than eight drugs were retained. Subsequently, Spearman rank-correlation coefficients were calculated for these pairwise similarities. Case-by-case inspection was performed to corroborate the concordance between KRDQN predictions and existing clinical evidence.

## 5. Summary

In summary, KRDQN offers substantial advantages in performance, interpretability, and generalizability for ADR prediction. Continued efforts in data curation, imbalance mitigation, and model refinement will further strengthen its utility in pharmacovigilance and clinical decision-making.

## Figures and Tables

**Table 1:** Comparison of baselines.

	AUC	Accuracy	Precision	Recall	F1
GCN	0.7590	0.8452	0.9058	0.5423	0.6784
DEEPWALK	0.7529	0.7759	0.7224	0.6778	0.6489
LSTM	0.7856	0.6892	0.7814	0.6321	0.6924
KPRN	0.8012	0.6935	0.7925	0.6487	0.6872
KRDQN	0.8327	0.7629	0.7372	0.8171	0.7751

**Table 2:** Comparison of mean fidelity values across baselines.

	Target_Mean	Pathway_Mean	Gene_Mean
DeepWalk	0.6025	0.3156	0.1861
Node2Vec	0.6973	0.5469	0.3839
GNN	0.6392	0.4315	0.5094
KRDQN	−1.2801	−0.4429	−0.6451

## Data Availability

The original data presented in the study are openly available in DrugBank (https://www.drugbank.com), Reactome (https://reactome.org), Pathway Commons (https://www.pathwaycommons.org) and ADReCS-Target Database.
